# Investigating the dynamics of *Plasmodium falciparum* gametocyte carriage in expectant women under intermittent preventive treatment with sulfadoxine-pyrimethamine in Kilifi, study protocol

**DOI:** 10.12688/openreseurope.19356.2

**Published:** 2025-04-11

**Authors:** Patience Kerubo Kiyuka, Mark Muricho, Nelson Ouma, Charles Muiruri, Amek Nyaguara, Martin Rono, Isabella Oyier, Mainga Hamaluba

**Affiliations:** 1 Center for Geographic Medicine Research Coast, Kenya Medical Research Institute, Kilifi, Kilifi County, 80108, Kenya; 2 KEMRI Wellcome Trust Research Programme, Kilifi, 80108, Kenya

**Keywords:** malaria, Plasmodium falciparum, sulfadoxine-pyrimethamine, intermittent preventive treatment, expectant mothers, pregnant women

## Abstract

**Introduction:**

Malaria in pregnancy remains a public health problem. The World Health Organization (WHO) recommends intermittent preventive treatment with sulfadoxine-pyrimethamine (IPTp-SP) to all pregnant women in moderate to high malaria transmission areas. Kenya's Ministry of Health recommends at least three doses of IPTp-SP (IPTp-SP3 +) to pregnant women in regions where malaria is endemic. Although SP remains cost-effective and effective for IPTp, there are two main challenges with the use of SP: i) widespread use of SP can lead to an increase in the prevalence of drug resistance molecular markers, including those encoding for *Plasmodium falciparum* dihydrofolate reductase ( *dhfr*) and P *f* dihydropteroate synthase ( *dhps*) and ii) SP, used either for curative or preventive treatment, is associated with microscopic and sub microscopic gametocytaemia, both of which contribute to sustained malaria transmission. Our study aims to investigate the dynamics of *Plasmodium falciparum* gametocyte carriage in pregnant women under intermittent preventive treatment with sulfadoxine-pyrimethamine in Kilifi.

**Methods:**

This will be a cross-sectional study and will recruit (N=462) expectant women attending antenatal care (ANC) clinics in four health facilities within the Kilifi Health and Demographic Surveillance Sites: Njunju, Pingilikani, Ngerenya, and Kilifi County Teaching and Referral Hospital (KCTRH). To be recruited into our study, women will need to be in their first or second pregnancy when they are more likely to have malaria and should have had at least one dose of sulfadoxine-pyrimethamine.

**Expected application of results:**

Our study will provide information on the current status of malaria during pregnancy in Kilifi and the prevalence of gametocytes among expectant mothers on IPT-SP. The results of this study may help inform new interventions to prevent malaria during pregnancy, including adding a third drug to SP with probable gametocytocidal effects.

## Plain language summary

Malaria during pregnancy poses significant risks to both maternal and fetal health, increasing the likelihood of maternal anemia, low birth weight, and premature delivery. In regions with high malaria transmission, such as Kilifi, Kenya, intermittent preventive treatment with sulfadoxine- pyrimethamine (IPTp-SP) is used to prevent malaria in pregnant women. Although IPTp-SP is generally regarded as safe and effective, recent evidence indicates that its performance may be suboptimal. This study aims to determine the prevalence of malaria parasitemia among pregnant women attending antenatal care (ANC) clinics in Kilifi. It will evaluate the IPTp-SP efficacy by determining parasite prevalence and gametocyte carriage in pregnant women. Additionally, the study will identify factors that influence IPTp-SP uptake by analyzing data from a previous larger study, the Kilifi Perinatal and Maternal (KIPMAT) study, which investigated the impact of various risk factors—such as HIV infection, maternal nutrition, malaria, and anemia—on maternal and infant morbidity and mortality. The findings from this research will help inform strategies to optimize malaria prevention and treatment during pregnancy, ultimately improving maternal and child health outcomes.

## Introduction/background

Malaria in pregnancy is a public health problem. According to the World Health Organization (WHO), 34% of pregnant women in the Africa region experienced malaria in 2023. Among the WHO subregions, the highest prevalence of malaria exposure during pregnancy was found in West Africa, while the lowest rates were observed in East and Southern Africa ( WHO, 2024). Malaria infection during pregnancy poses a substantial risk to the mother, fetus and newborn. The consequences of malaria in pregnancy include maternal anaemia, low birth weight, pre-term birth, stillbirth, and infant and maternal deaths ( McGregor *et al.*, 1983). In areas of stable malaria transmission, adults, including pregnant women, do acquire immunity to malaria and can, therefore, control but not clear malaria infections ( Takem & D'Alessandro, 2013). There are three strategies to combat malaria in pregnancy: intermittent preventive treatment during pregnancy (IPTp), insecticide-treated bed nets and prompt diagnosis and effective treatment. 'IPTp constitutes the administration—irrespective of parasitemia—of a curative treatment dose, usually sulfadoxine-pyrimethamine (SP) at predefined intervals, during routine antenatal care' ( Mockenhaupt *et al.*, 2008).

The WHO recommendation on IPTp-SP has undergone revisions. In 2004, the recommendation was a minimum of two doses of IPTp-SP during pregnancy. In 2012, the recommendation was changed to at least three doses of IPTp-SP. This was a result of supporting evidence that three doses of sulfadoxine-pyrimethamine were associated with higher birth weight and lower risk of low birth weight than the standard 2-dose regimens ( Kayentao *et al.*, 2013).In Kenya, the practice is the administration of three IPTp-SP doses (IPTp-SP3+) to pregnant women in regions where malaria is endemic. Over the last decade, most countries in sub-Saharan Africa have widely adopted IPTp-SP policies for pregnant women. However, there are wide disparities in utilizing the highly cost-effective IPTp-SP intervention. In a large cross-sectional analysis of 18,603 women (aged 15–49 years) from 8 African countries, the prevalence of 3 doses of IPTp-SP varied from 60% in Ghana to 31% in Sierra Leon ( Yaya *et al.*, 2018). In Kenya, the latest data indicate that the uptake of more than 3 doses of SP during pregnancy stands at 43% ( Miatu *et al.*, 2024). Some of the factors associated with the uptake of antimalarial drugs among pregnant women include the timing of ANC initiation, number of ANC visits, income level, a woman's educational level and stock-outs of the drugs ( Ameh *et al.*, 2016).

*Plasmodium falciparum* transmission from the human host to the mosquito requires the presence of infectious gametocytes in the human peripheral blood. Indeed, the probability that a mosquito will be infected depends, among other factors, on the duration, prevalence and density of gametocyte carriage in the human host ( Drakeley *et al.*, 1999). Highly effective antimalarial drugs target to restrain gametocyte carriage and reduce disease progression. Gametocyte carriage and infectivity to mosquitoes are consistently higher in drug-resistant parasites than in drug-sensitive malaria parasites, which fuels the spread of antimalarial resistance. This is because slower clearance and prolonged presence of asexual parasites associated with resistance increase gametocytogenesis ( Barnes & White, 2005). Of all the antimalarial drugs ever used, sulfadoxine-pyrimethamine has been associated with the highest prevalence and density of gametocyte carriage ( Targett *et al.*, 2001).

Reports of increasing resistance to SP are now threatening the effectiveness of IPTp-SP. Polymorphisms in the *P. falciparum* dihydrofolate reductase/dihydropteroate synthetase ( *Pfdhfr/Pfdhps*) genes are known to be associated with high SP resistance and treatment failure, with parasites having the K540E mutation (a proxy for the *Pfdhfr*– *Pfdhps* quintuple mutant) linked with resistance rendering SP ineffective in clearing *Pf* infections ( Desai *et al.*, 2016). On the other sextuple mutant parasites, quintuple mutant plus the additional *Pfdhps* A581G mutation is associated with reduced IPTp-SP efficacy ( Naidoo & Roper, 2013).

A most recent study analysis of SP resistance over the last 25 years in Kilifi observed that the quintuple mutation form of sulfadoxine-pyrimethamine resistance witnessed a sustained increase over the years up to 83.6% in 2018 ( Omedo *et al.*, 2022). However, the Omedo *et al.* study analysed samples from young children; therefore, it is unknown whether SP mutant parasites are also on the increase in pregnant women who continue receiving SP and that warranty investigation. There are conflicting data on the risks and benefits of IPTp-SP. Previous studies indicate that even if there is a rise in the prevalence of molecular markers of resistance among pregnant women, SP can effectively reduce the malaria burden during pregnancy and extend protection to non-malarial cases of low birthweight ( Taylor *et al.*, 2012). However, the SP, used for curative or preventive treatment, is associated with microscopic and sub-microscopic gametocytaemia, contributing to sustained malaria transmission ( Bousema & Drakeley, 2011). Furthermore, SP provides intermittent clearance or suppression of existing asymptomatic infections from the placenta and can prevent new infections for several weeks by maintaining suppressive drug levels ( Karunajeewa *et al.*, 2009). It is this prolonged clearance period for SP that drives gametogenesis.

Our study aims to understand the dynamic of gametocyte carriage in pregnant women under intermittent preventive treatment with sulfadoxine-pyrimethamine, specifically if the varying doses of SP influence gametocyte carriage. We will also assess SP resistance markers in women under IPTp-SP. To comprehensively understand the determinants of IPTp-SP uptake, we will utilize a dataset from a previous study, the Kilifi Perinatal and Maternal (KIPMAT) study. Previously, the data was used to assess the impact of IPTp-SP on birth outcomes ( Kamau *et al.*, 2023). In the present study, we will apply the logistic regression model to evaluate predictors of uptake of optimal doses of IPTp-SP.

### Rationale/justification

In malaria-endemic countries, the WHO recommends that pregnant women receive Intermittent Preventive Treatment with sulfadoxine-pyrimethamine (SP) during their antenatal care clinics ( WHO, 2014). SP works through two modes of action: i) treatment effect by clearing existing asymptomatic infections in the placenta and ii) prophylactic effect in which suppressive drug levels are maintained in the placenta for some time, and this prevents new infections from occurring ( White, 2005). IPTp-SP intervention faces two main challenges that hinder its effectiveness: first, emergence and widespread resistance to SP, and second, sub-optimal uptake of the intervention. Across various malaria epidemiological settings in Africa, there have been reports of polymorphisms in the *P. falciparum* dihydrofolate reductase/dihydropteroate synthetase ( *Pfdhfr/Pfdhps*) genes, which are associated with a high-level of SP resistance ( Amimo *et al.*, 2020). Furthermore, parasites containing quintuple mutations, which is a combination of three *dhfr* mutations (N51I, N59R, and S108N) and two *dhps* mutations (A437G and K540E) and is a predictor of treatment failure have also been reported ( Kublin *et al.*, 2002). Although the percentage of women under IPTp-SP has increased over the years, uptake of the intervention has remained low ( Yaya *et al.*, 2018), The latest WHO World Malaria report indicates that although coverage of IPTp1, IPTp2 and IPTp3 increased, from 54%, 45% and 34% in 2021, to 64%, 54% and 42% in 2022, respectively, it fell short of the target of 80% ( World Malaria Report, 2023). Individual-level factors and healthcare challenges have been attributed to the varying IPTp-SP uptake ( Berchie *et al.*, 2024).

Kenya revised its guidelines to include IPTp3 doses around 2015 ( Kamau *et al.*, 2023). In a large study examining around 27,000 deliveries along Kenya's coastal region, an IPTp-SP dose response protection against low birth weight and stillbirths was observed ( Kamau *et al.*, 2023). Nevertheless, SP is associated with a high prevalence and density of gametocyte carriage. Therefore, our study proposes understanding the relationship between gametocyte carriage and the doses of SP among pregnant women in Kilifi. Additionally, we will assess the determinants of IPTp-SP intervention within our settings. Previous studies on SP mutations in Kenya have primarily focused on children ( Omedo *et al.*, 2022), with limited attention given to malaria in pregnancy.

### Null hypothesis

IPTp-SP does not drive gametocyte carriage in pregnant women.

### Study aim

Investigating the prevalence of *Plasmodium falciparum*, drug resistance and gametocyte carriage in women under intermittent preventive treatment with sulfadoxine-pyrimethamine.

### Specific objectives

1) Determine the prevalence of *Plasmodium falciparum* infections in pregnant women.
2) Determine the prevalence of *Pf* gametocyte carriage and its association with IPTp-SP doses in pregnant women in Kilifi
3) Analyse the relationship between IPTp-SP doses and the frequency of SP-resistant parasites.

### Secondary objectives

a) To understand the factors affecting the uptake of three or more doses of IPTp-SP in Kilifi.
b) Analyse SP resistance markers in expectant women under IPTp-SP

## Methods

### Study design

This will be a cross-section study to understand the dynamic of gametocyte carriage in women under IPTp-SP within Kilifi County.

### Study location

The Kenya Medical Research Institute, Centre for Geographic Medicine Research Coast (KEMRI CGMRC), will conduct the study. The study participants will be recruited from three dispensaries: Junju, Ngerenya, Pingilikani, and KCH Hospital. KCH is urban, while the other health facilities are located in rural settings. Recruitment will take place between February and August 2025.

### Study population

The aim is to recruit expectant women presenting at ANC clinics at KCH, Pingilikani, Ngerenya and Junju and within the Kilifi Demographic Health Surveillance Sites.

Study inclusion criteria

1) Women in their first or second pregnancies- who are likely to be infected with malaria due to their limited acquisition of immunity to malaria.
2) Should have had at least one dose of SP

Study exclusion criteria

- Expectant women with no documented evidence of SP dosage in their ANC booklet
- Participants who refuse to consent
- Women with obstetric complications
- Expectant women who are on malaria drugs or have been treated for malaria in the 2 weeks prior to the current ANC visit
- Pregnant women on co-trimoxazole prophylaxis

### Sampling and recruitment procedures

Women who meet our study inclusion criteria and reside within the Kilifi Health Demographic and Surveillance Site will be recruited for our study. We will recruit participants from four health facilities: KCTRH, Ngerenya, Pingilikani and Junju. We will recruit expectant women during routine ANC visits at the four health facilities. We will approach and consent eligible participants after the attending nurse or clinician has seen them. The KEMRI CGMRC has had a long-standing engagement with these health facilities. As part of the study engagement activities prior to study imitation, we will request that the health facility allocate us a small room or space to set up a temporary room so we can confidentially consent our study participants and for other study-related activities. Since one of our eligibility criteria is that the expectant women should have had at least one dose of SP and the current guidelines state that "provision of SP should be done for above 13 weeks' of gestation", we anticipate recruiting women who will be attending their second or more ANC visits when standard antenatal care profile procedures (blood group, HB measurements, malaria test, blood glucose, syphilis, and urine protein etc) will have already been done. We cannot use this routine blood from the antenatal care profile procedures. However, if the participant is required to undergo an RDT malaria test as part of their routine care, we will only request an additional 0.35ml, collected using a fingerpick, from them if they turn RDT positive. We will collect blood for the RDT and the additional 0.35ml (if malaria-positive) only after the participant completes routine checks with the attending nurse. We anticipate that study participants will take approximately 1 hour to participate in our study procedures. Blood samples from study participants will undergo tests listed in Table 1 below. We will collect the following demographic data from eligible study participants: mother's age, marital status, education level, parity status (based on parity and gravida), number of ANC attendance and number of doses of malaria prophylaxis.

**Table 1.** Sample collection and laboratory procedures.

Goal	Laboratory procedures	Location	Volume of blood required
Rapid malaria detection	RDT	To be done at the ANC clinic	50ul
Presence or absence of malaria parasites	Microscopy (gold standard)	Additional blood samples will be shipped to KEMRI- CGMRC laboratories	50ul
Gametocyte carriage	qPCR		200ul
Detection of resistance to SP markers	DNA extraction, Sanger/NGS sequencing		100ul
Total blood volume			400 ul (0.4ml)

### Sample size

The minimum required base sample size using individual randomization ( Daniel, 1999):


$$n\geq \frac{Z_{\alpha /2}^{2}P(1-P)}{d^{2}}Deff$$

With P being the expected prevalence of malaria in the study area (in proportion to one) and d the required level of precision taken to be half of P here. Assuming a 7.1% malaria prevalence based on a previous study on malaria prevalence among adults aged 15–49 years in Kilifi ( Kamau *et al.*, 2020), we get a sample of size *n* = 202 pregnant women, which after increasing by a design effect factor of 2 and 15% to take care of contingencies gives 475.

*Sample distribution across the study sites*

Kilifi County exhibits heterogenous malaria transmission

- Low transmission: Ngerenya (North) and Kilifi town
- Moderate transmission: Pingilikani and Junju (South) ( Kimenyi *et al.*, 2022; Mogeni *et al.*, 2016).

Given that gametocyte carriage varies with transmission intensity, age and recent malaria episodes and SP use ( Jafari-Guemouri *et al.*, 2018; Muthui *et al.*, 2019; Stepniewska *et al.*, 2008) and IPTp-SP use is uniform across the county, we will recruit proportionally from all sites (Pingilikani, Junju, Ngerenya, and Kilifi County hospital) until the target sample size (n = 475) is reached.

To understand the factors affecting the uptake of three or more doses of IPTp-SP in Kilifi, we will analyze surveillance data from the Kilifi Perinatal and Maternal (KIPMAT) study ( Seale *et al.*, 2015). The KIPMAT study was established at the Kilifi County Hospital in 2011 to standardize admission procedures to improve the clinical care for expectant women ( Seale *et al.*, 2015). It was a clinical surveillance study of pregnant women and their newborns. The study was embedded into the routine clinical care as a way of supporting and improving clinical care in resource limited setting. At admission for labour, all mothers were registered by trained fieldworkers. Data were collected on residential location, age, education level, gravidity, HIV status, ANC visits and SP frequency. At delivery, fieldworkers recorded information on delivery outcomes, sex, gestational age and infant weight. Data were saved onto the My Structured Query Language (MySQL) database.

### Laboratory procedures

***Microscopy for asexual and gametocyte quantification.*** Thick and thin blood films will be taken from eligible expectant women participants. The thin blood films will be fixed with 100% methanol and stained with 3% Giemsa stain for 45 minutes before being examined for parasites. Thick films will be air-dried before staining. Two independent readers will determine the malaria parasite and gametocyte presence or absence; a third reader will resolve discordant readings.

***RNA extraction.*** 200 µL of whole blood from each study participant will be stored (1:5) in 1000 µL RNA Protect Cell Reagent (Qiagen, Australia) at −80 °C until RNA extraction. RNA extraction will be performed on the whole sample using RNeasy Plus Mini Kit (Qiagen, Australia) following the manufacturer's instructions with the treatment of DNase on-column digestion using RNase-Free DNase set (Qiagen, Australia) to eliminate genomic DNA. 100 µL of RNA extract will be eluted.

***qPCR quantification of gametocyte carriage.*** qPCR will be performed using an in-house protocol. Sex-specific qRT-PCR assays will measure RNA transcripts specific to female and male gametocytes. For example, we will target the *Plasmodium falciparum* gametocyte surface protein (pfs25) mRNA as the female marker and the *P. falciparum* PF3D7_1469900 mRNA transcript a recently characterized gametocyte-enriched transcript ( *Pf*MGET) as the male marker, Table 2 below lists our expanded panel of primer probes. All qRT-PCR assays will be conducted with a One-Step RT-PCR mix (Qiagen, Australia) using methods previously described ( Collins *et al.*, 2018) with optimization and validation using cultures P. falciparum gametocytes. Our in-house controls ensure specificity and sensitivity for detecting gametocyte markers. Briefly, 0.45 µM of each primer and 0.18 µM of Taqman probe will be used in each PCR reaction. Amplification will be performed in Applied Biosystems QuantStudio [model number QuantStudio TM 5-96 well 0.2ML Block) Real-Time PCR System under the following cycling conditions: 50 °C reverse transcription for 30 min, 95 °C incubation for 15 min, followed by 45 cycles of 95 °C for 15 s and 60 °C for 60 s. Internal control available in-house will be used to quantify the gametocyte carriage.

**Table 2.** Expanded primer probe panel for the qPCR assay.

Target	Gene	Primer/Probe Sequences
**AP2-G** (commitment)	PF3D7_1222600	F: GGAAAGATACGACAAGAGGACAT R: CCATTCTCAATGCACCATCATTAC Probe: FAM-CCCTGTATACCCTTCTTCGAAAGCATGC-MGB
**SBP1** (both male and female)	PF3D7_0501300	F: CCGACGAACCAACACAATTAC R: ACTAGCTGCTTCTCCAACTAAA Probe: JOE-TTGGCCGAAGTAGTTTCGGATGCA-MGB
**GEXP5** (female)	PF3D7_0936600	F: AGTGCTGAGGATAAGGATTCATT R: CACCACTTCTCAAACAACCACT Probe: Texas Red-AGCTGTCTGAATTAGTAGACTCCA-MGB
**CCP4** (novel female)	PF3D7_0903800	F: CACATGAATATGAGAATAAAATTG R: TAGGCGAACATGTGGAAAG Probe: FAM-AGCAACAACGGTATGTGCCT-MGB
**P25** (female)	PF3D7_1031000	F: TAATGCGAAAGTTACCGTGGA R: ACAGTCTTTTCGTCACATTTCAG Probe: JOE-TCAGATGAGTGGTCATTTGGA-MGB
**PfMGET** (male)	PF3D7_1469900	F: CGGTCCAAATATAAAATCCTG R: TGATTCATTTTCTTGCTTCAGA Probe: VIC-CAGCTCCAGCATTAAAAACACA-MGB

***DNA extraction and SP resistance testing.*** Resistance to SP has been linked to point mutations in the parasite dihydrofolate reductase ( *dhfr*) and dihydropteroate synthase ( *dhps*) genes, which we will analyse. The *dhfr* mutation confers resistance to pyrimethamine ( Lozovsky *et al.*, 2009), while *dhps* confer resistance to sulfadoxine and other sulpha drugs. Parasite genomic DNA will be extracted from EDTA blood samples using commercially available DNA Qiagen kits, *dhfr* and dhps fragments will be amplified using in-house nested PCR sequence protocols. Nested purified PCR products will be sequenced using a Big Dye Terminator v3.1 cycle sequencing kit. The reactions will be precipitated in 70 % ethanol to clean up dye terminators, rehydrated in 10 ul HiDi formamide and then sequenced on a 3130 ABI Genetic Analyzer. Table 1 indicates a summary of laboratory procedures.

### Ethics statement

 Approval for the study was obtained from the Kenya Medical Research Institute (KEMRI) Scientific Ethics Review Unit (SERU) with the reference number KEMRI/SERU/5071 on November 14, 2024. A research license from the National Commission for Science, Technology, and Innovation was granted on December 6, 2024, under (Ref No. 503343). Additionally, the Kilifi County Department of Health Services approved the study to be conducted in Kilifi County on December 16, 2024. We will seek written informed consent from expectant mothers who are eligible to participate in this study.

### Data storage

Data will be collected in the field using electronic handheld devices. All data will be backed up daily on a remote server within the Kilifi site and the secondary server in Nairobi.

### Data management

Data collected for this study will be part of the larger dataset at the CGMR-C, which links clinical, laboratory, and demographic databases and is written in MySQL software https://www.mysql.com, an open source database. This system is managed centrally, and this study will use existing data managers who will assist in conducting data checks and providing datasets when needed by the study investigators.

### Statistical analysis

Participant socio-demographic characteristics will be summarised by numbers and percentages if categorical. Continuous data will be summarised by mean, standard deviation (SD), and range if data are normally distributed and median, interquartile range (IQR) and range if data are skewed. We will summarise the prevalence of *Plasmodium falciparum* infections and Pf gametocyte carriage in pregnant women, together with the associated 95% Agresti-Coull confidence interval. The Chi-square test will compare prevalence across groups (such as size and other characteristics). Regression analysis will be used to investigate factors affecting the uptake of three or more doses of IPTp-SP in Kilifi. Data will be analysed in version 17.0 (Stata Corp, College Station, TX), and graphs will be generated using R ( R Core Team, 2024). Furthermore, we will describe the relationship between IPTp-SP doses and the frequency of SP-resistant parasites.

### Ethical considerations

We will conduct our study according to the ethical considerations outlined in the Helsinki Declaration.

***Risk to participants.*** The risks to participating subjects will be those associated with fresh blood collection, including temporary local pain and possibly mild bruising. We will minimize risks to study participants by using well-trained staff to obtain blood collection from study participants. No additional blood collection will be required from those found to be RDT-negative. Participants will be approached and requested to participate in our study at the reception as they wait to be seen by the attending nurse. A request to collect an initial 0.05ml will only be made if the attending clinician has not requested an RDT malaria test. An additional blood collection of 0.35ml will be requested if the expectant woman has RDT-positive test results. We will ask for these samples (0.05ml for RDT and an additional 0.35ml if RDT positive) after the mother has completed her routine checks with the attending nurse. In addition, participation in the study will be voluntary, and participants will not incur extra costs for participating as they will be selected from routine ANC attendance. We anticipate study participants taking approximately one hour in our study procedures. We will offer refreshments to all the mothers who participate in our study.

***Benefits to study subjects/community.*** There are no direct benefits to those involved in this study. It is hoped that this research will result in new knowledge that will lead to the development of alternative approaches to the prevention and treatment of malaria during pregnancy and that will be of broader benefit to all societies affected by the disease. For women with RDT-positive tests, this information will be shared immediately with the attending nurse/clinician to guide their care and management.

***Informed consent.*** We will obtain written informed consent from eligible study participants. Participants will be approached and requested to participate in the study at the reception area as they wait to see the attending nurse. We will distinguish standard routine procedure from the additional consent sought for this study. We will explain that should the expectant women decline to participate in our study, the availability of health care in the dispensary or hospital will remain the same. They will be explained that they are free to decline to participate in the study should they change their mind mid-way into consenting to the study or at any point during the study procedures.

For those who consent to participate in our study, after they complete their routine checks, and if the clinician has not requested a malaria test, we will request an RDT test. An additional blood sample will be ordered from those with malaria parasites. This will involve the inconvenience of a further blood sample. We will use trained fieldworkers for consenting. All malaria episodes will be managed per standard practice at the designated dispensary or hospital.

***Community engagement.*** A CAST (Community Engagement Advice for Studies) group will be set up to support the planning and implementation of engagement activities. CASTs are study-specific groups set up by the Community Liaison Manager in CGMRC and includes members of the Community Liaison Group and core members of the study team. The CAST group will advise throughout the study on community engagement, including strategic planning and responding to any emerging issues during the study. The CAST group will also be able to support similar activities at the participating health facilities.

We will seek to engage the Sub-County Health Managers for the four participating health facilities (Ngerenya, Junju, Pingilikani and KCTRH) at the onset of the study. Before beginning the study, we will also contact the hospital and dispensary committee, the staff at ANC, and other community leaders within the targeted facilities. We will provide regular updates when required. Furthermore, at the end of the study, we will communicate and share the study results with participants and communities at all the study sites. Results will be shared at a population level, not per everyone's outcome.

We are aware (from our previous community engagement activities) that studies among pregnant women can be sensitive, especially where blood is drawn -and for our case, more than once if RDT is positive)- as such, key messages will be carefully developed to address any community concerns during the engagement. Our fieldworkers will also work with the healthcare workers to participate in the routine health talks. The fieldworkers will use the health talk platform to mention our study activities in the participating health facilities.

***Methods for maintaining confidentiality.*** Data will be stored on password-protected servers in the KEMRI-CGMRC. Datasets shared with collaborators for analysis will be de-identified, i.e. not include participants' names but rather a study number).

***Data sharing and protection.*** In the future, de-identified information collected or generated during this study may be used to support new research by other researchers in Kenya and other countries interested in understanding malaria in pregnancy. In all cases, we will only share information with other researchers in ways that do not reveal individual participants' identities following appropriate permissions. Any future research using information from this study must first be approved by a local (including the data governance committee) or national ethics committee.

The data from this study will undergo strict quality control before it is released for local analysis or external sharing. The cleaned data will be made available to study investigators. Priority will be given to access to the data analysis and publication to the study investigators before the data can be made publicly available. Study participants will have appropriately been informed about safeguarding confidentiality should their data be shared with external researchers after the study—requests for data from external investigators after publication can be made through the PI. If permission to use data is granted, the use will be compatible with the informed consent forms used to collect the data. The study site will keep personally identifiable information about participants from this study for seven years after the study has finished under applicable Data Protection Requirements in Kenya. Study participants have the right to access the personal data we hold that pertains to them and to object to or make corrections to the processing of all or part of the personal data.

### Timelines for proposed study activities

We anticipate starting study engagement activities in quarter (Q) one of 2025 and thereafter begin participant recruitments. Details of the study activities timeframe are listed in Table 3 below

**Table 3.** Study activities timeframe.

Activity	Year 1		Year 2	
	Q1-2	Q3-4	Q1-2	Q3-4
Protocol development and approval				
Study engagement activities				
Data collection				
Data analysis				
Report writing and manuscript preparations				

***Study limitations.*** Our study will use Sanger sequencing platform. Sanger sequencing offers various advantages. It is a cost-effective and fast way for reading sequences of small targeted genomic regions and it remains the gold standard for accurate detection of single nucleotide variants. Sanger sequencing has been widely used to describe *Plasmodium falciparum* dihydrofolate reductase ( *Pfdhfr*) and dihydropteroate synthase ( *Pfdhps*) gene mutations associated with sulfadoxine/pyrimethamine resistance ( Chauvin *et al.*, 2015; Guémas *et al.*, 2023). However, one of the limitations of Sanger sequencing platform unlike next generation sequencing, is that we are more likely to miss out on minor variants and mixed infections. However, for this instance we are interested in understanding SP resistance where the markers for resistance are well defined, We are collecting a total of 0.4ml of blood and we will use 200ul for RNA extraction. In a sample with low parasitemia, the genetic material will be below the detection levels required for downstream analysis. For example, in instances of placental malaria the peripheral parasite genetic material will be too low to adequately quantify gametocyte carriage. Nevertheless, we will use RNA extraction kits that are well optimized to capture even low quantity parasite genetic material.

## Data Availability

No data are associated with this article. Harvard Dataverse: Replication Data for: Dynamics of Plasmodium falciparum gametocyte carriage in pregnant women under intermittent preventive treatment with sulfadoxine-pyrimethamine and its resistance in Kilifi, study protocol, DOI: https://doi.org/10.7910/DVN/JV8E9M ( Kiyuka, 2024) This project contains the following extended data: CRF_IPTp-SP protocol.docx Facility ICF_for_IPTp-SP protocol.docx Data are available under the terms of the Creative Commons Zero "No rights reserved" data waiver (CC0 1.0 Public domain dedication). This study adheres to the Strengthening the Reporting of Observational studies in Epidemiology (STROBE) guidelines for cross-sectional studies. Harvard Dataverse: Replication Data for: Dynamics of Plasmodium falciparum gametocyte carriage in pregnant women under intermittent preventive treatment with sulfadoxine-pyrimethamine and its resistance in Kilifi, study protocol, DOI: https://doi.org/10.7910/DVN/JV8E9M ( Kiyuka, 2024) This project contains the following reporting guidelines STROBE checklist. Data are available under the terms of the Creative Commons Zero "No rights reserved" data waiver (CC0 1.0 Public domain dedication).
