# Supplementary material for: Investigating the dynamics of Plasmodium falciparum gametocyte carriage in expectant women under intermittent preventive treatment with sulfadoxine-pyrimethamine in Kilifi, study protocol
Source: Open Res Eur. 2025 Apr 11;5:60. Originally published 2025 Mar 3. [Version 2] doi: 10.12688/openreseurope.19356.2 (PMC11933784; doi:10.12688/openreseurope.19356.2)
Supplement: Peer review history

# Reviewer report: Reviewer response for version 1

**DOI:** 10.21956/openreseurope.20949.r51822

**Published:** 2025-04-02

**Authors:** Liusheng Huang

**Affiliations:** 1 University of California San Francisco, San Francisco, California, USA

General comments:

 Parasitemia during pregnancy is common and presents a significant risk to birth outcomes. IPTp-SP has been widely used during pregnancy to prevent malaria and improve birth outcomes. However, the efficacy of SP as IPTp is compromised due to the emergence of drug resistance. This study protocol addresses two important questions: sulfadoxine-pyrimethamine resistance and gametocyte carriage.

 Specific objective 4 could be “to evaluate association between IPTp-SP doses and gametocyte carriage in pregnant women in Kilifi, alternatively, update objective 2:  Determine the prevalence of Pf gametocyte carriage and its association with IPTp-SP doses in pregnant women in Kilifi.

 Study inclusion criteria:

 “Women in their first or second pregnancies- who are likely to be infected with malaria.”  How to determine “likely to be infected with malaria” ?  participants in endemic region? Family members have malaria?

 Specific comment:

 Page 6, qPCR quantification of gametocyte carriage: …..we will use target the Plasmodium falciparum gametocyte surface protein (pfs25)….. Please revise the sentence: “We will use… or we will target…” it is unclear.

Is the study design appropriate for the research question?

Yes

Is the rationale for, and objectives of, the study clearly described?

Yes

Are sufficient details of the methods provided to allow replication by others?

Yes

Are the datasets clearly presented in a useable and accessible format?

Not applicable

Reviewer Expertise:

Antimalarial pharmacokinetics and bioanalysis.

I confirm that I have read this submission and believe that I have an appropriate level of expertise to confirm that it is of an acceptable scientific standard.

# Response

**Published:** 2025-04-03

**Authors:** Patience Kiyuka

**Affiliations:** Biosciences, Kenya Medical Research Institute, Nairobi, Nairobi County, Kenya

*Specific objective 4 could be “to evaluate association between IPTp-SP doses and gametocyte carriage in pregnant women in Kilifi, alternatively, update objective 2:  Determine the prevalence of Pf gametocyte carriage and its association with IPTp-SP doses in pregnant women in Kilifi.*

**Response** We agree with the reviewer and will update objective 2 to read: Determine the prevalence of *Pf* gametocyte carriage and its association with IPTp-SP doses in pregnant women in Kilifi *Study inclusion criteria:*

*“Women in their first or second pregnancies- who are likely to be infected with malaria.”  How to determine “likely to be infected with malaria” ? Participants in endemic region? Family members have malaria?*

**Response:** We apologize for the confusion this statement may have caused as we were referring to the acquisition of immunity due to the argument that; During pregnancy, the parasites can sequester to the placenta by binding to chondroitin sulfate A (CSA), a process mediated by members of a large, diverse protein family called P. falciparum erythrocyte membrane protein 1 (PfEMP1) protein variants (Nunes & Scherf, 2007).  Studies have shown that women acquire immunity to Pf malaria over successful pregnancies due to the acquisition of antibodies against the CSA-binding parasite (Duffy, 2007), with prevalence and severity higher in multigravida women than primigravida women (Touré et al., 2019).   We will change that statement to read. Women in their first or second pregnancies- who are likely to be infected with malaria due to limited acquisition of immunity to malaria.  

*Page 6, qPCR quantification of gametocyte carriage: …..we will use target the Plasmodium falciparum gametocyte surface protein (pfs25)….. Please revise the sentence: “We will use… or we will target…” it is unclear.*  

**Response** Corrected to, we will target the *Plasmodium falciparum gametocyte surface protein (pfs25)*  

**Acknowledgement** We thank the reviewer for their meticulous critique, which has strengthened the rigor and clarity of our work. All changes will be implemented in the revised manuscript.

# Reviewer report: Reviewer response for version 1

**DOI:** 10.21956/openreseurope.20949.r51824

**Published:** 2025-03-24

**Authors:** Roland Ibenipere Funwei

**Affiliations:** 1 Babcock University,, Ilishan-Remo, Ogun, Nigeria

The study is well conceptualized and executed. The title and abstract are well written. However, most of the tenses used are future tenses. Could this be justified?

 2. "In 2021, approximately 40 million pregnant women were at risk of *Plasmodium falciparum* malaria infection ( WHO Malaria Report, 2021)". Why 2021 and not 2024 WHO report? Is there no difference in the epidemiology of malaria in pregnant women between 2021 and 2024? If there is, then cite the most recent report.

 3. "( *pfdhfr/pfdhps*)" should be *Pfdhfr/Pfdhps.*

*4. *It could have been interesting to analyze the relationship between IPTp-SP doses and gametocyte carriage as a fourth objective.

Is the study design appropriate for the research question?

Yes

Is the rationale for, and objectives of, the study clearly described?

Yes

Are sufficient details of the methods provided to allow replication by others?

Yes

Are the datasets clearly presented in a useable and accessible format?

Yes

Reviewer Expertise:

Malaria chemotherapy, antimalarial drug efficacy, genomics, and surveillance of resistant parasites.

I confirm that I have read this submission and believe that I have an appropriate level of expertise to confirm that it is of an acceptable scientific standard.

# Response

**Published:** 2025-04-01

**Authors:** Patience Kiyuka

**Affiliations:** Biosciences, Kenya Medical Research Institute, Nairobi, Nairobi County, Kenya

*1. The study is well conceptualized and executed. The title and abstract are well written. However, most of the tenses used are future tenses. Could this be justified?* As this is a publication of the study protocol, we used future tense to describe what we intend to do. *2. "In 2021, approximately 40 million pregnant women were at risk of Plasmodium falciparum malaria infection (* *WHO Malaria Report, 2021* *)". Why 2021 and not 2024 WHO report? Is there no difference in the epidemiology of malaria in pregnant women between 2021 and 2024? If there is, then cite the most recent report.* We have cited the most recent WHO malaria report of 2024 as follows: According to the World Health Organization (WHO), 34% of pregnant women in the Africa region experienced malaria in 2023. Among the WHO subregions, the highest prevalence of malaria exposure during pregnancy was found in West Africa, while the lowest rates were observed in East and Southern Africa(WHO, 2024).   3 *. "(pfdhfr/pfdhps)" should be Pfdhfr/Pfdhps.* Corrected *pfdhfr/pfdhps to Pfdhfr/Pfdhps* *4.It could have been interesting to analyze the relationship between IPTp-SP doses and gametocyte carriage as a fourth objective.* We thank the reviewer for this suggestion. We will include the fourth objective as: To analyze the relationship between IPTp-SP doses and gametocyte carriage **Acknowledgement** We thank the reviewer for their meticulous critique, which has strengthened the rigor and clarity of our work. All changes will be implemented in the revised manuscript.

# Reviewer report: Reviewer response for version 1

**DOI:** 10.21956/openreseurope.20949.r51820

**Published:** 2025-03-11

**Authors:** Adebanjo Adegbola

**Affiliations:** 1 Obafemi Awolowo University, Ile Ife, Nigeria

A clearly defined research idea and a well-structured study protocol monitoring the presence or absence of gametocytes after treatment with IPTp-SP. The research findings will help address a key issue regarding the use of SP for chemoprevention.. 

**Major concern**

**Sample size**

 (1) I do not quite follow the calculation for the sample size. From the sample size determination, it is clear how the authors arrived at 202 and the use of design effect of 2 to make a sample size of 404. However, the estimation for drop-out is not clear. If a 15% drop out is foreseen, the expected sample size should be 404/(1-0.15) = 404/0.85 = 475, instead of 462 provided in the manuscript. For instance, [462 - 0.15*462 = 393; compared with 475 - 0.15*475 = 404.

 (2) Authors need to provide the sample size distributed to each study site

**qPCR quantification of gametocyte carriage**

 (1) According to the authors, this is an in-house protocol; has this protocol been optimised or validated to detect and quantify Pf gametocytes?

 (2) The authors need to clarify whether they are adopting the Collins et al. (2018) method or using another in-house developed method for the qRT-PCR gametocyte protocol.

**Ethics and Consent**.

 On page 9, I suggest deleting this section, as it has already been adequately addressed in another part.

**Minor Correction**

**Introduction**

 (1) Paragraph 2, Line 2, IPTP-SP should be written as IPTp-SP

 (2) Paragraph 5, line 4 Increase should be written increase.

**Laboratory Procedures**

**DNA extraction and SP resistance testing**

 (1) ' *Dhfr*' should be written ' *dhfr*'

 (2) The authors need to confirm the volume of HiDi formamide used, '10 ml' or '10 ul'

**Benefits to study subjects/community**

 this study instead of "these studies"

**Study limitations**

 Line 10, to adequately **quantify...... **instead of to adequately **quantity**.........

Is the study design appropriate for the research question?

Yes

Is the rationale for, and objectives of, the study clearly described?

Yes

Are sufficient details of the methods provided to allow replication by others?

Yes

Are the datasets clearly presented in a useable and accessible format?

Yes

Reviewer Expertise:

Malaria Research: Antimalarial Efficacy Studies, Malaria molecular surveillance, Genomics and bioanalytical assays.

I confirm that I have read this submission and believe that I have an appropriate level of expertise to confirm that it is of an acceptable scientific standard.

# Response

**Published:** 2025-03-18

**Authors:** Patience Kiyuka

**Affiliations:** Biosciences, Kenya Medical Research Institute, Nairobi, Nairobi County, Kenya

**1 Sample size**  

(1) I do not quite follow the calculation for the sample size. From the sample size determination, it is clear how the authors arrived at 202 and the use of design effect of 2 to make a sample size of 404. However, the estimation for drop-out is not clear. If a 15% drop out is foreseen, the expected sample size should be 404/(1-0.15) = 404/0.85 = 475, instead of 462 provided in the manuscript. For instance, [462 - 0.15*462 = 393; compared with 475 - 0.15*475 = 404.

*(2) Authors need to provide the sample size distributed to each study site*

***Responses*** **Sample size calculation**

 We sincerely thank the reviewer for identifying the error in our initial calculation. As noted, accounting for a 15% dropout rate requires adjusting the sample size as follows. Adjusted sample size = 404/(1-0.15) = 475 We will correct this in the revised manuscript.

**Sample distribution across study sites:** Kilifi County exhibits heterogeneous malaria transmission

- Low transmission: Ngerenya (North) and Kilifi town
- Moderate transmission: Pingilikani and Junju (South) (Kimenyi et al., 2022; Mogeni et al., 2016).

Given that gametocyte carriage varies with transmission intensity, age and recent malaria episodes and SP use (Jafari-Guemouri et al., 2018; Muthui et al., 2019; Stepniewska et al., 2008) and IPTp-SP use is uniform across the county, we will recruit proportionally from all sites (Pingilikani, Junju, Ngerenya, and Kilifi County hospital) until the target sample size (n = 475) is reached.  

**2. qPCR quantification of gametocytes**

*(1) According to the authors, this is an in-house protocol; has this protocol been optimised or validated to detect and quantify Pf gametocytes?*

*(2) The authors need to clarify whether they are adopting the Collins et al. (2018) method or using another in-house developed method for the qRT-PCR gametocyte*

***Responses*** **Protocol validation** We apologize for the confusion. The qRT-PCR protocol is adapted from Collins et al (2018), with optimization and validation using cultured *P. falciparum* gametocytes. Our in-house controls ensure specificity and sensitivity for detecting gametocyte markers.

 Furthermore, we expanded the primer-probe panel to include targets for male and female gametocytes, as detailed below:  

**Target** **Gene** **Primer/Probe Sequences** **AP2-G** (commitment) PF3D7_1222600 F: GGAAAGATACGACAAGAGGACAT

 R: CCATTCTCAATGCACCATCATTAC

 Probe: FAM-CCCTGTATACCCTTCTTCGAAAGCATGC-MGB **SBP1** (both male and female) PF3D7_0501300 F: CCGACGAACCAACACAATTAC

 R: ACTAGCTGCTTCTCCAACTAAA

 Probe: JOE-TTGGCCGAAGTAGTTTCGGATGCA-MGB **GEXP5** (female) PF3D7_0936600 F: AGTGCTGAGGATAAGGATTCATT

 R: CACCACTTCTCAAACAACCACT

 Probe: Texas Red-AGCTGTCTGAATTAGTAGACTCCA-MGB **CCP4** (novel female) PF3D7_0903800 F: CACATGAATATGAGAATAAAATTG

 R: TAGGCGAACATGTGGAAAG

 Probe: FAM-AGCAACAACGGTATGTGCCT-MGB **P25** (female) PF3D7_1031000 F: TAATGCGAAAGTTACCGTGGA

 R: ACAGTCTTTTCGTCACATTTCAG

 Probe: JOE-TCAGATGAGTGGTCATTTGGA-MGB **PfMGET** (male) PF3D7_1469900 F: CGGTCCAAATATAAAATCCTG

 R: TGATTCATTTTCTTGCTTCAGA

 Probe: VIC-CAGCTCCAGCATTAAAAACACA-MGB  

1. **Ethics and Consent.**

*On page 9, I suggest deleting this section, as it has already been adequately addressed in another part.*

***Responses*** The redundant ethics section will be removed, as requested

1. **Minor Corrections**

***Responses***

1. **Introduction**

- Corrected “IPTP-SP” to **“IPTp-SP”** (p. 2, line 4).
- Changed “Increase” to **“increase”** (p. 5, line 4).

1. **Laboratory Procedures**

- Corrected *'Dhfr' to **dhfr***
- Clarified HiDi formamide volume as **10 µl** (not 10 mL)

1. **Benefits to community**

- Revised “these studies “ to **“this study”**

1. **Study limitations**

- Corrected “quantity” to “ **quantify” **(line 10)

**Acknowledgements**

 We thank the reviewer for their meticulous critique, which has strengthened the rigour and clarity of our work. All changes will be implemented in the revised manuscript.   References Jafari-Guemouri, S., Dhiab, J., Massougbodji, A., Deloron, P., & Tuikue, N. N. (2018). Dynamics of Plasmodium falciparum gametocyte carriage in pregnant women under intermittent preventive treatment with sulfadoxine–pyrimethamine in Benin. *Malaria Journal*, *17*(1), 356. https://doi.org/10.1186/s12936-018-2498-8 Kimenyi, K. M., Wamae, K., Ngoi, J. M., de Laurent, Z. R., Ndwiga, L., Osoti, V., Obiero, G., Abdi, A. I., Bejon, P., & Ochola-Oyier, L. I. (2022). Maintenance of high temporal Plasmodium falciparum genetic diversity and complexity of infection in asymptomatic and symptomatic infections in Kilifi, Kenya from 2007 to 2018. *Malaria Journal*, *21*(1), 192. https://doi.org/10.1186/s12936-022-04213-7 Mogeni, P., Williams, T. N., Fegan, G., Nyundo, C., Bauni, E., Mwai, K., Omedo, I., Njuguna, P., Newton, C. R., Osier, F., Berkley, J. A., Hammitt, L. L., Lowe, B., Mwambingu, G., Awuondo, K., Mturi, N., Peshu, N., Snow, R. W., Noor, A., … Bejon, P. (2016). Age, Spatial, and Temporal Variations in Hospital Admissions with Malaria in Kilifi County, Kenya: A 25-Year Longitudinal Observational Study. *PLoS Medicine*, *13*(6), e1002047. https://doi.org/10.1371/journal.pmed.1002047 Muthui, M. K., Mogeni, P., Mwai, K., Nyundo, C., Macharia, A., Williams, T. N., Nyangweso, G., Wambua, J., Mwanga, D., Marsh, K., Bejon, P., & Kapulu, M. C. (2019). Gametocyte carriage in an era of changing malaria epidemiology: A 19-year analysis of a malaria longitudinal cohort. *Wellcome Open Research*, *4*, 66. https://doi.org/10.12688/wellcomeopenres.15186.2 Stepniewska, K., Price, R. N., Sutherland, C. J., Drakeley, C. J., von Seidlein, L., Nosten, F., & White, N. J. (2008). Plasmodium falciparum gametocyte dynamics in areas of different malaria endemicity. *Malaria Journal*, *7*(1), 249. https://doi.org/10.1186/1475-2875-7-249
